# The Role of Inorganic-Organic Bio-Fillers Containing Kraft Lignin in Improvement in Functional Properties of Polyethylene

**DOI:** 10.3390/ma14092114

**Published:** 2021-04-22

**Authors:** Karol Bula, Łukasz Klapiszewski, Adam Piasecki, Teofil Jesionowski

**Affiliations:** 1 Institute of Materials Technology, Faculty of Mechanical Engineering, Poznan University of Technology, PL-60965 Poznan, Poland; 2 Institute of Chemical Technology and Engineering, Faculty of Chemical Technology, Poznan University of Technology, PL-60965 Poznan, Poland; teofil.jesionowski@put.poznan.pl; 3 Institute of Materials Engineering, Faculty of Materials Engineering and Technical Physics, Poznan University of Technology, PL-60965 Poznan, Poland; adam.piasecki@put.poznan.pl

**Keywords:** lignin, inorganic-organic hybrid materials, bio-fillers, polyethylene

## Abstract

In this study, MgO-lignin (MgO-L) dual phase fillers with varying amounts of lignin as well as pristine lignin and magnesium oxide were used as effective bio-fillers to increase the ultraviolet light protection and enhance the barrier performance of low density polyethylene (LDPE) thin sheet films. Differential scanning calorimetry (DSC) was used to check the crystalline structure of the studied samples, and scanning electron microscopy (SEM) was applied to determine morphological characteristics. The results of optical spectrometry in the range of UV light indicated that LDPE/MgO-L (1:5 wt/wt) composition exhibited the best protection factor, whereas LDPE did not absorb ultraviolet waves. Moreover, the addition of hybrid filler decreased the oxygen permeability factor and water vapor transmission compared with neat LDPE and its composites with pristine additives, such as lignin and magnesium oxide. The strong influence of the microstructure on thin sheet films was observed in the DSC results, as double melting peaks were detected only for LDPE compounded with inorganic-organic bio-fillers: LDPE/MgO-L.

## 1. Introduction

Presently, material science, which covers polymer materials, is searching for benefits associated with the wide application of bio-additives in traditional thermoplastics. The benefits are usually connected with the improvement in technological as well as practical properties. Numerous considered applications of bio-additives are focused on the modification of polymers used for packaging [1,2,3]. This narrow declaration is connected with the processing window, which is above 200 °C for engineering polymers and thus limits the use of bio-based cellulose-like particles.

Widely used and commercially available high-volume polymer matrices include, e.g., high density polyethylene (HDPE), low density polyethylene (LDPE), polypropylene (PP), polystyrene (PS), poly(lactide) (PLA), poly(3-hydroxybutyrate) (PHB), polybutylene adipate-co-terephthalate (PBAT), etc., whereas biopolymers processed using a low temperature regime that are ready to use as bio-additives mainly include cellulose and lignocellulose [4,5,6,7,8,9,10,11]. A comprehensive review of the possible lignin interactions in more than twenty polymeric systems was published by Kun et al. [12]. In general, due to the highly hydrophilic nature of lignin and other lignocellulosic fillers, they should undergo modification or plasticization to improve their dispersion in polymeric materials, or a compatibilizer should be added to increase interfacial adhesion between the components. Notably, lignin (aminated lignin) can also be used as a component for absorbers of the electromagnetic field with a maximum absorbing capacity at 8 GHz band [13]. Moreover, lignosulfonate was successfully used as a dispersant for improving the fluidity of the cement paste with an additional increase in compressive strength [14].

If lignin is considered as a functional filler in thermoplastics, its influence on rheological and anti-aging properties has to be determined. One of the most expected outcomes resulting from the presence of lignin is the gain of antioxidation properties. Almost two decades ago, Pouteau et al. introduced lignin as an antioxidant agent in polypropylene [15]. They examined the oxidation effect based on oxidation induction time (OIT) at 180 °C. They found that the OIT value for PP blended with organosolv lignin was 20 times longer (670 min) than for pristine PP (30 min). The oxidation behavior was related to the average aggregate surface area of lignin particles as well as total OH content, whereas the induction time decreased roughly with OH content. They also confirmed that lignosulfonate has no effect on OIT due to its great polarity. The same positive impact of lignin on thermo-oxidative durability was noted for the LDPE polymer [16]. The influence of lignin on the thermal degradation of isotactic polypropylene, investigated by thermogravimetric analysis, was reported by Canetti et al. [17]. An increase in thermal degradation temperature of the blends was observed under oxidative and non-oxidative conditions. The increase was noticeable for the experiments carried out in air atmosphere, where the interactions between the polypropylene and the lignin led to the formation of a protective surface able to reduce the oxygen diffusion toward the polymer bulk. Antioxidant effectiveness in relation to PP was detected by Gregorova et al. [18]. Another positive influence of using lignin on polymer properties should be underlined in the case of permeability reduction, which was confirmed in systems with PLA [19].

Some of the published results also showed lignin activity in UV absorption, such as how PLA films blended with lignin exhibited absorption in nearly the entire UV wavelength range (200–700 nm) [20]. Absorption of UV light spectra is mainly linked with the phenolic groups and conjugated carbonyl groups present in lignin [21]. Lignin, as an ultraviolet absorbent, was positively evaluated by Toh et al. [22] and by Domenek et al. [23].

A positive aspect of lignin is associated with the increase in thermal degradation temperature of PP and its flame retardation properties [17,24]. The fire retardancy of lignin is also increased by small amounts of other known non-halogenated fire retardants to reduce the flammability of PP; hence, synergism can be achieved [25,26]. 

Thinking about lignin as a polymer modifier, we assumed that not only functional properties such as permeability, UV stabilization, and flame retardation should be strictly taken into account in direct applications. From a practical point of view, much attention should also be paid to technological properties. Therefore, in our previous studies, we confirmed the positive role of kraft lignin combined with oxides in film weldability and strength of welded sheets [27]. We also showed that the MgO-lignin hybrid additive gained the thermoforming ability of low density polyethylene in case of uniform wall thickness distribution of thermoformed shapes [28].

Since we proved that dual-phase fillers may be used as an effective modifier of technological as well as mechanical properties of polymers, it is obligatory to investigate their impact on some crucial properties adequate for film packaging materials. In this research, compounds based on low density polyethylene with MgO-lignin hybrid materials were studied to validate their potential availability as packaging materials by means of gas permeation and ultraviolet barrier properties. By presenting the newly obtained results, the material characteristics of LDPE/MgO-lignin hybrid compounds were determined. Therefore, we decided to update the already published results and complete the data regarding technological and application material properties, which are crucial from a practical point of view.

## 2. Materials and Methods

### 2.1. Materials

The following reagents were used to conduct the experiments: (*i*) magnesium oxide–MgO (CAS Number: 1309-48-4), type caustic calcined magnesia (CCM) with purity ≥ 99% from Sigma Aldrich (Steinheim am Albuch, Germany), and (*ii*) kraft lignin–alkali lignin (CAS Number: 8068-05-1), with an average M_w_ ~10,000 from Sigma Aldrich (Steinheim am Albuch, Germany).

The polymer matrix used in this work was a low density polyethylene (LDPE) Malen E FGNX 23-D006 grade, from Basell Orlen Polyolefins (Płock, Poland). The producer describes this grade as being suitable for manufacturing of highly transparent, very fine films. Moreover, extruded films exhibit very good mechanical properties and are notable for their high transparency and gloss. Malen E FGNX 23-D006 has a melt flow rate (MFR) of 0.8 g/10 min at 190 °C. As a polar component and a compatibilizing agent, polyethylene-graft-maleic anhydride copolymer was used, supplied by Sigma-Aldrich (Steinheim am Albuch, Germany), with 0.5% maleic anhydride content.

### 2.2. Preparation of MgO-Lignin Dual Bio-Fillers

MgO-lignin dual bio-fillers were synthesized via grinding and mechanical-alloying methods. This process was described in detail in our previous works [25,26].

As part of this publication, MgO-lignin systems with the following weight ratio of inorganic to organic parts were prepared: 5:1, 1:1, and 1:5. All important details regarding the dispersive and morphological characteristics, scanning electron microscopy images, and physicochemical results were presented and discussed in our previous paper [27]. Additionally, MgO particle diameter data are presented as follows: d(0.1)–0.6 µm, d(0.5)–1.2 µm, and d(0.9)–2.2 µm, which means that the percentage contribution (10, 50, 90%, respectively) of the particles volume distribution is below this diameter. The average particle size of MgO is 1.5 µm. 

### 2.3. Preparation of LDPE/PE-g-MAH/MgO-Lignin Composites 

**Figure 1:**
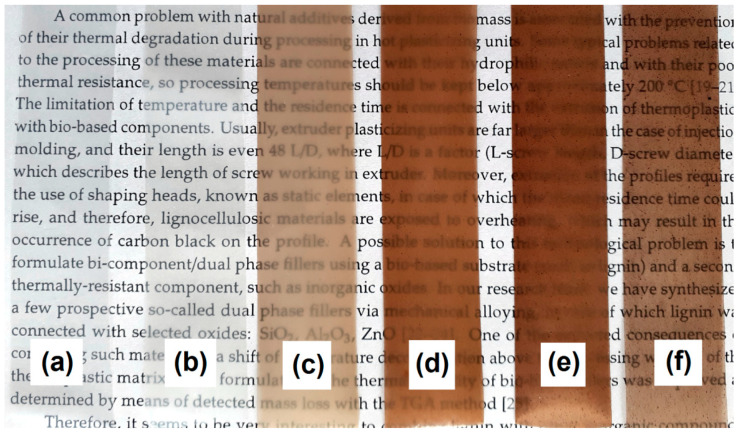
The apparent transparency cutaway foil strips of extruded samples: (**a**) pristine LDPE, (**b**) LDPE/MgO, (**c**) LDPE/MgO-L (5:1 wt/wt), (**d**) LDPE/MgO-L (1:1 wt/wt), (**e**) LDPE/MgO-L (1:5 wt/wt), and (**f**) LDPE/Lignin.

The compounding of LDPE/PE-g-MAH and the prepared MgO-lignin fillers with different lignin amounts in the filler structure was carried out by melt mixing in a twin screw extruder Zamak 16/40 EHD (Zamak Mercator Sp. zo.o., Skawina, Poland), working in a co-rotating mode. The plasticizing unit, which includes screws with the diameter of 16 mm, is characterized by a 40 L/D ratio and is equipped with three segments of kneading disc blocks, which facilitate higher shear stress. All composites were extruded with a barrel temperature of 160–185 °C and a screw rotation speed of 150 rpm. Some detailed information regarding screw configuration and other processing parameters were presented in our previous work [29]. The pellets of LDPE and granulated composites of LDPE/PE-g-MAH/MgO-lignin were cast-extruded using a single screw extruder (Metalchem 28/30, Gliwice, Poland) (screw diameter of 28 mm, and 30 L/D ratio) and a semi-laboratory chill-roll device (Remi-Plast, Czerwonak, Poland). The line was equipped with a 1 mm thick and 170 mm long slit die and two chill rolls. The temperature profile at the plasticizing unit was set to 160, 175, 165, 174, 180, and 190 °C from feed to die, with a main screws rotation speed of 70 rpm. For all compositions, the chill roll temperature was set to 40 °C using an external cooling chiller. During thin film casting, only one chill roll speed was used: 2 m/min. The optical pictures of foil strips are presented in Figure 1. The contributions of all components in prepared composites are listed in Table 1. 

We decided to present all further material characteristics only for the compound with 5 wt % filler content and film sheets produced with a chill roll speed of 2 m/min. This results from all of the technological and material properties that we already published being established exactly for the compounds defined above.

### 2.4. Differential Scanning Calorimetry (DSC) Analysis

The melting and crystallization temperatures as well as heats of fusion and crystallization of the samples were measured using a Netzsch 204 F1 Phoenix (Selb, Germany) differential scanning calorimeter. Three runs were applied for sample characteristics. The samples were first heated to 250 °C at a rate of 10 °C/min, then isothermally treated for 5 min to eliminate their thermal history and subsequently cooled to 25 °C at a rate of 10 °C/min, under nitrogen atmosphere. In the first heating segment, the samples showed thermal effects that originate from oriented structure due to stretching in the chill-roll device. The second endotherm was recorded in the same regime as first one. The heat of crystallization was calculated relevant to that of pure crystalline PE (293 J/g according to [30,31]).

### 2.5. Microstructural Investigations

The morphology of the film surfaces was observed using SEM (Tescan Mira3, Tescan, Brno, Czech Republic) equipped with EDS (Oxford Instruments Ultim Max 65). An accelerating voltage of 12 kV was applied. Prior to SEM observation, the sheet surface of selected films was cleaned with methanol and dried for 220 min at 60 °C. All samples were sputtered coated with an amorphous layer of carbon with a thickness of approximately 20 nm. The Jeol JEE 4B vacuum evaporator (Peabody, MA, USA) was used. The contents of elements such as Mg were analyzed. Due to the low atomic number of carbon, its content was not measured. The distribution of element concentrations in the form of EDS patterns maps were performed. 

### 2.6. UV-Vis Absorption Test

The optical properties of the composite films were determined by measuring the absorption of light at 190–1100 nm, at the optical resolution of 4 nm, using a UV-Vis spectrophotometer (UV line 9400, Schott Instruments, Mainz, Germany). 

### 2.7. Gas Permeability and Water Vapor Permeation Tests

Oxygen permeability (OP) of films was measured using a Lyssy L100-5000 analyzer (Systech Illinois, Devens, MA, USA), following the manometric method (pressure change via gas transmission through films), according to the PN-EN ISO 2556:2002 standard. Films were conditioned for at least 2 days at 23 °C and 52% RH prior to analysis. Their thicknesses were measured by a micrometer and varied from 96–255 µm. Three individual tests were conducted for each sample. The OP is given in the units (mL/m^2^/24 hours) at a pressure difference of 100 kPa. 

Water vapor permeability (WVP) was assessed according to standard PN-EN ISO 15106-1:2005 by using a Lyssy L80-5000 (Systech Illinois, Devens, MA, USA) analyzer. The equipment can measure high permeability materials using special sample cards. Pristine and modified LDPE films were conditioned for at least 2 days at 23 °C and 52% RH prior to analysis. Their thicknesses were measured by a micrometer and varied from 60–247 µm. Samples were cut and sealed in aluminum foil with a round shape open area of 5 cm^2^. The WVP of the samples was measured at 38 °C and at a relative humidity of 90%. Three individual tests were carried out for each ample.

## 3. Results and Discussion

### 3.1. Thermal Behavior of the LDPE/MgO-Lignin Composites

**Figure 2:**
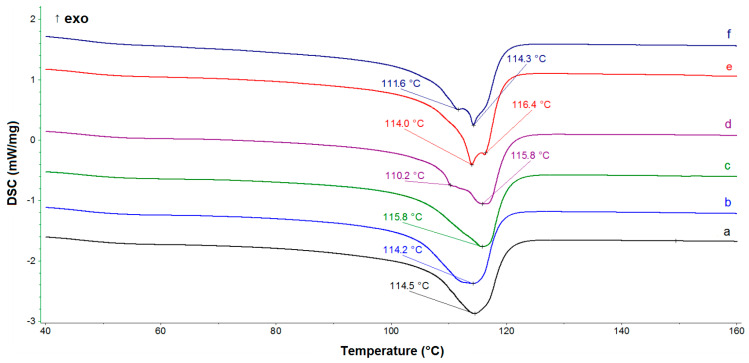
The DSC curves obtained during the first heating of (a) pristine LDPE, (b) LDPE/MgO, (c) LDPE/MgO-L (5:1 wt/wt), (d) LDPE/MgO-L (1:1 wt/wt), (e) LDPE/MgO-L (1:5 wt/wt), and (f) LDPE/lignin.

**Figure 3:**
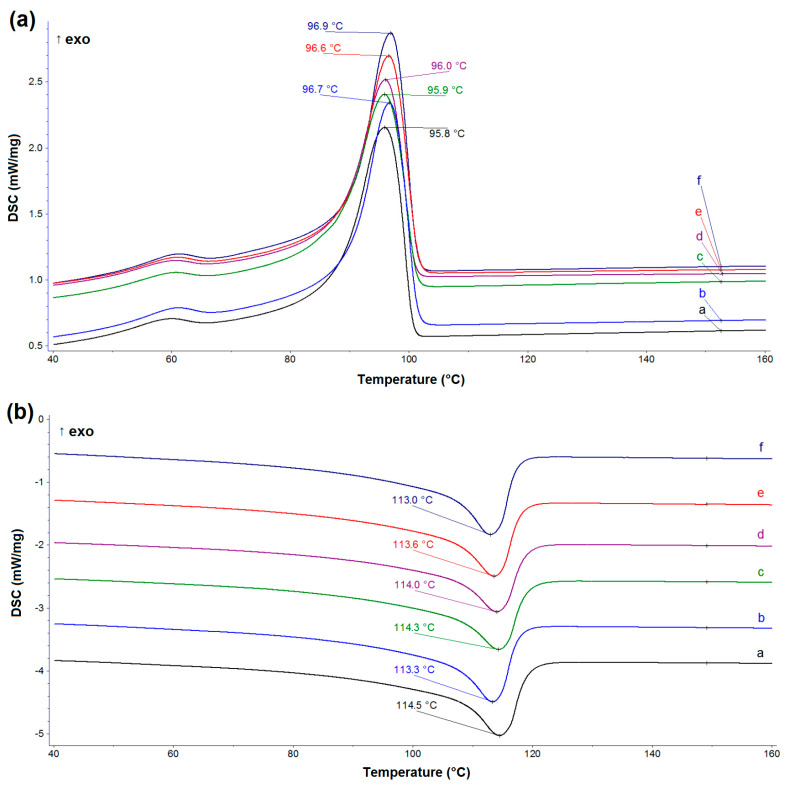
(**a**) The DSC curves obtained during the cooling of (a) pristine LDPE, (b) LDPE/MgO, (c) LDPE/MgO-L (5:1 wt/wt), (d) LDPE/MgO-L (1:1 wt/wt), (e) LDPE/MgO-L (1:5 wt/wt), and (f) LDPE/lignin. (**b**) The DSC curves obtained during the second heating.

The DSC curves recorded during the first heating (Figure 2) show differences in the melting region of LDPE. It can be clearly seen that the thermograms of samples with dual fillers and pristine lignin include double melting peaks, shifted by approximately 2–3 °C. For LDPE/MgO and LDPE/MgO-L (5:1 wt/wt) samples (blue- and green-colored curves, respectively), there is a slight indication of shoulder peak, located on the main melting effect, however, without curve inflection. The separation of the main melting peak into two closely placed peaks (overlapping effect) probably resulted from the interaction of particles, as lignin was doped and arrangement of molecular chains located close to filer particles originated from stretching in the chill-roll device. Notably, double melting peaks are present for composites with higher lignin content. Cheng et al. [32] stated that an addition of nano-microparticles would involve two crystalline morphologies with crystalline regions: homogenous nucleated and heterogenous nucleated. Another possibility for the appearance of the two melting peaks could be associated with the presence of the compatibilizer–malleated polyethylene (MAPE). As shown by Diop et al. [33], a possible bonding reaction between lignin and MAPE resulted in high interface adhesion. The authors confirmed their theoretical considerations based on the morphology investigation, which brought evidence that lignin was perfectly bonded to the matrix. By the incorporation of low molecular weight proadhesive agent MAPE into a nonpolar (LDPE)–polar (lignin) system, the interface tension was reduced and some molecular rearrangement during film stretching was possible. If we consider only the relationship between componential ratios (oxide/lignin), it is clear that increasing the amount of lignin in hybrid fillers results in an increase in crystallinity of LDPE and the appearance of a double peak of endothermic peak appeared (Table 2). The crystallization temperature from the melting state was characterized by identical values for all samples, which means that under controlled cooling conditions in the DSC chamber, without stretching, the tested fillers had a negligible influence on the crystallization process and crystallinity (Figure 3a). As shown in Figure 3b, based on the presented curves adequate for second heating, only one main endothermic effect appeared for all tested samples. Moreover, the crystallinity of LDPE and its composites seemed to be constant, without regular relation to the sample composition, and varied between 37.5% and 41.3% (Table 2). A similar relation of thermal properties was observed by Olmos et al. during studies of LDPE combined with silica fumes [34]. Additionally, during cooling after isothermal treatment at 250 °C, the crystallization process was similar for all samples.

### 3.2. Microscopic Observation

**Figure 4:**
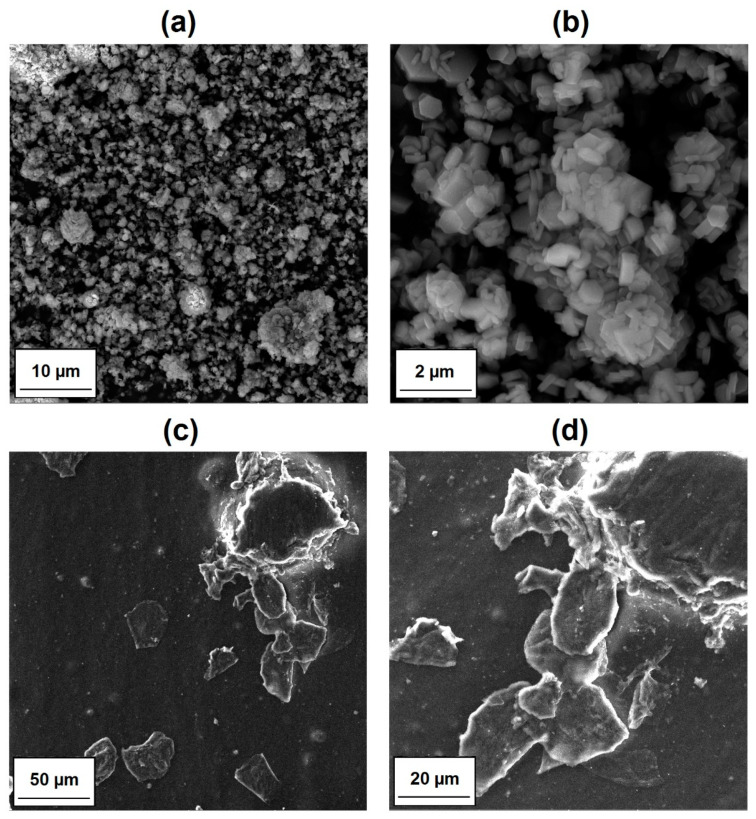
Representative SEM images of (**a**,**b**) MgO filler particles and (**c**,**d**) LDPE/MgO composite films.

**Figure 5:**
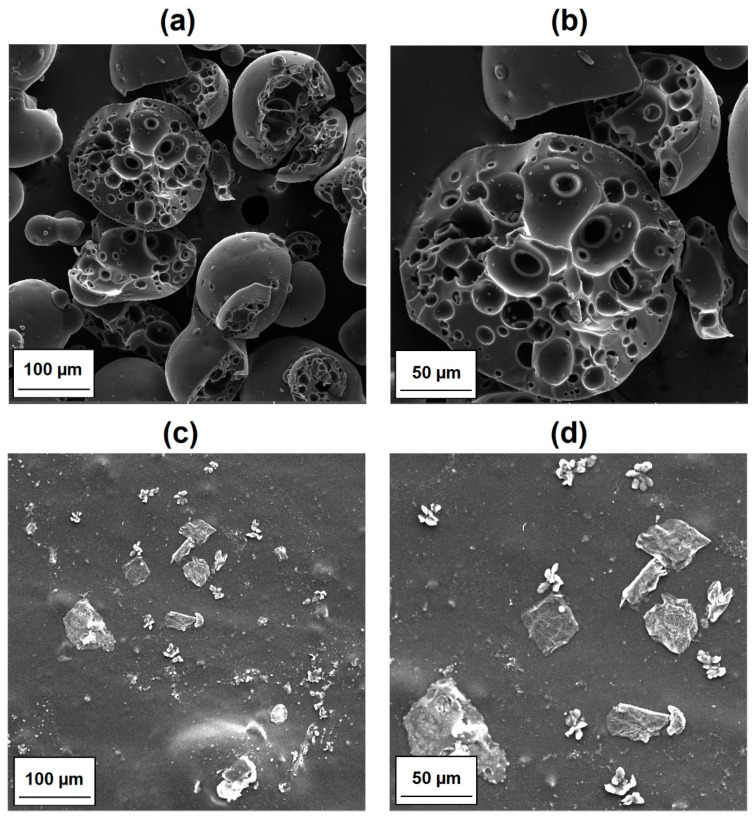
Representative SEM images of (**a**,**b**) lignin filler particles and (**c**,**d**) LDPE/lignin composite films.

**Figure 6:**
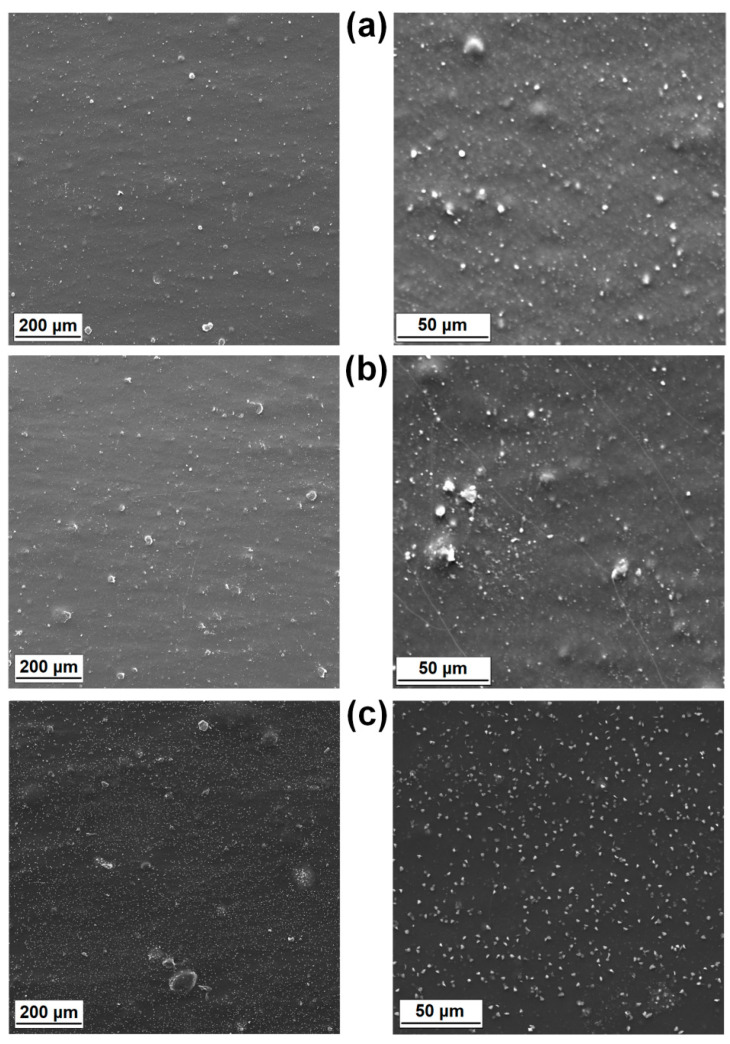
Representative SEM images of composite films: (**a**) LDPE/MgO-L (5:1 wt/wt), (**b**) LDPE/MgO-L (1:1 wt/wt), and (**c**) LDPE/MgO-L (1:5 wt/wt).

**Figure 7:**
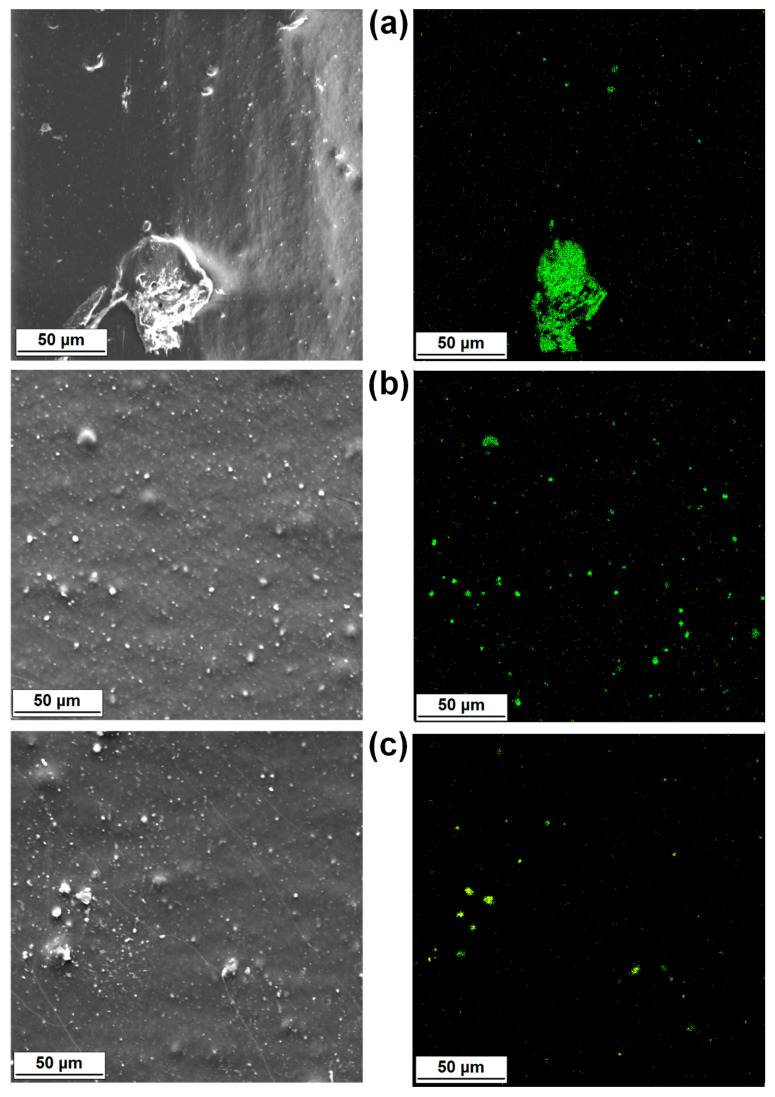

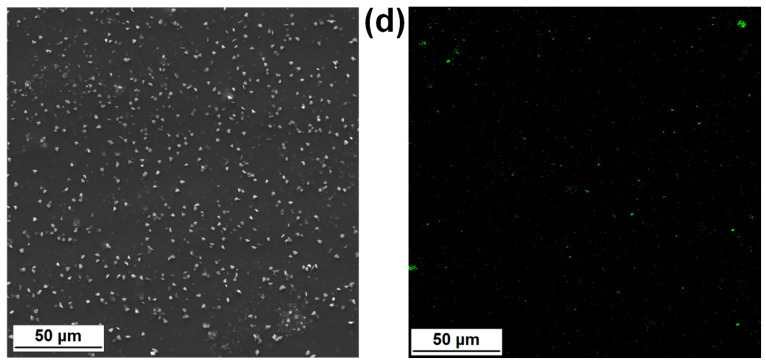
Representative SEM-EDS images of magnesium mapping: (**a**) LDPE/MgO, (**b**) LDPE/MgO-L (5:1 wt/wt), (**c**) LDPE/MgO-L (1:1 wt/wt), and (**d**) LDPE/MgO-L (1:5 wt/wt).

The SEM micrographs of the MgO and lignin particles as well as images of the surface morphology of their composites with LDPE are shown in Figure 4 and Figure 5, respectively. Pristine MgO exhibited a tendency to form agglomerated structures, which were composed of platy or disc-shaped particles (Figure 4a,b). The morphology of LDPE/MgO also included platy inclusions (particles covered by the polymer layers), but the isolated platy forms were characterized by a diameter of 20 μm. In turn, kraft lignin particles possessed a rather spherical shape, with numerous clearly visible microbubbles inside one single broken particle (Figure 5a,b). Partially flat, embedded particles can be seen in Figure 5c,d, which confirm the fragmentation of lignin particles during the melt processing, especially in the twin screw extruder equipped with kneading blocks. In contrast, a noticeably improved particle dispersion was seen for composites with hybrid fillers (MgO-L). Dispersed particles in those composites were separated, and only small, spread aggregates were visible (Figure 6a,b). The most uniform particle distribution and no visible aggregates were noted for composites with the MgO-L (1:5) hybrid filler. In Figure 6c, only isolated particles with diameter lower than 10 μm can be observed. This observation was confirmed by the magnesium Mg mapping using SEM-EDS analysis (Figure 7). In Figure 7a, there is a huge irregular inclusion in the morphology, and EDS analysis confirmed that it represented agglomerated magnesium oxide particles. As mentioned earlier, composites with hybrid fillers MgO-L (5:1) and MgO-L (1:1) exhibited a morphology with small agglomerates, which is also confirmed by the local, minor concentration of magnesium in polyethylene matrix. EDS mapping of the composite with MgO-L (1:5) hybrid filler revealed the uniform spread distribution of magnesium, without local clumps, confirming the observation using the SEM technique for this composite (Figure 7d).

### 3.3. UV Absorption Properties

**Figure 8:**
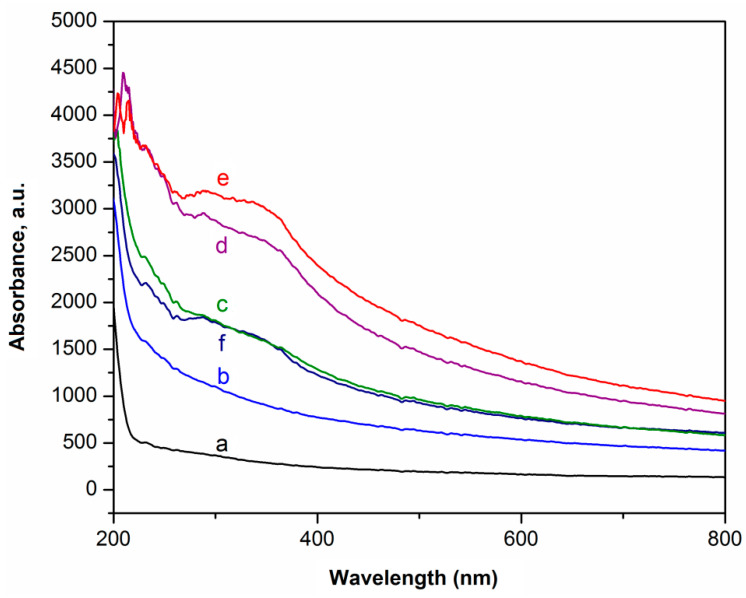
UV–Vis spectra of LDPE-based composite films: (a) pristine LDPE, (b) LDPE/MgO, (c) LDPE/MgO-L (5:1 wt/wt), (d) LDPE/MgO-L (1:1 wt/wt), (e) LDPE/MgO-L (1:5 wt/wt), and (f) LDPE/lignin.

The UV–visible light absorption curves of the composites in the wavelength range of 200–800 nm are presented in Figure 8. The neat LDPE film exhibited almost no absorption in the wavelength range greater than 220 nm due to the rather high transparency of the sample (Figure 8). Addition of pristine magnesium oxide into LDPE did not change the absorbance trace, as confirmed by a lack of any light absorption at wavelengths above 240 nm. The incorporation of the hybridized filler MgO-lignin system into LDPE, especially for dual fillers MgO-L (1:1 wt/wt) and MgO-L (1:5 wt/wt), resulted in a sharp absorption peak (located at approx. 220 nm) and a broad peak formed in the range of 280–400 nm. Shankar et al. also reported that PLA blended with lignin and silver nitrate exhibited two bands of UV absorbance, one narrow with a shoulder effect (lignin absorbance) and one broad at 450 nm, connected with silver action [20]. The first peak can be attributed to the presence of a chromophore C=O in the lignin chain, while the broad peak indicates the absorption resulting from phenolic groups and conjugated structure of lignin. It is unexpected that we did not observe a strong absorption signal and protection that might be associated with the presence of lignin. However, the broad peak in the LDPE/lignin material spectra was also present. This behavior can result from additional operations such as ball milling and sieving, which were only applied in the case for dual filler powders. Pristine magnesium oxide and lignin, as byproducts, did not undergo such treatment. These results indicated that the composite films with the highest amount of lignin incorporated into a hybridized filler labelled as a MgO-L (1:5 wt/wt) possessed an excellent UV-barrier property. The increased UV-barrier property of the mentioned material was primarily attributed to the absorption of UV light by aromatic groups and conjugated carbonyl groups in lignin [22,35,36] and to the very uniform spread of those particles (without huge aggregates) in the LDPE matrix, as was noted during the SEM investigation (Figure 6c).

### 3.4. Barrier Properties of Studied Compounds

Food packaging of polymeric films is the main application of the studied compounds, for which the barrier property of the film should be improved. The permeable properties of LDPE/MgO-lignin composites films were evaluated in terms of the oxygen transmission rate (OTR) and water vapor transmission rate (WVTR), and the results are presented in Table 3 and Table 4, respectively. The oxygen permeability value of the LDPE, which was equal to 779 mL/m^2^/day atm, is a similar value to that presented by Mooninta et al. (697 mL/m^2^/day atm) [37]. The addition of MgO filler resulted in a dramatic increase in the OTR of the film up to 1292.6 mL/m^2^/day atm, which was the highest result obtained in this investigation. For the majority of the tested samples, the OTR was also higher than in case of LDPE, which was attributed to the agglomerates of MgO (Figure 5a) and poor dispersion of lignin and some hybridized filler (Figure 6a–c). Surprisingly, the application of the MgO-L (1:5 wt/wt) hybrid additive decreased the oxygen transmission rate in LDPE by approx. 18%. The explanation of this beneficial aspect is associated with the very fine dispersion of the filler in the matrix, which promotes increased tortuosity of the permeate path, as frequently suggested in nanocomposite systems [38]. For the water vapor transmission test, in contrast with the gas barrier properties, the WVTR values of all tested composites were lower compared with that of neat LDPE. The reduction in WVTR was equal to at least 38% in case of MgO, as the additive with the worse dispersibility. In contrast, the higher reduction in water transition occurred in LDPE/MgO-L (1:5 wt/wt) and for LDPE/lignin systems, by 56% and 68%, respectively. This means that the increase in lignin content results in a better barrier for water transition. One of the possible explanations of this feature is the presence of –OH groups in lignin and the affinity to water molecules, which are able to form a hydrogen bond, and therefore reduction of water permeability increases. A similar observation was reported by Kaboorani et al. when LDPE was mixed with thermoplastic starch (TPS) and particles of cellulose nanocrystals (CNCs) [39]. In this work, the WVTR reduced from 22.2 to 17.2 (g/m^2^/day) for LDPE/TPS and LDPE/TPS/CNCs composites, respectively.

## 4. Conclusions

In the present study, we examined the functional properties of LDPE/MgO-lignin composites interpreted as barrier properties and ultraviolet light absorption efficiency. As shown, the oxygen permeability, which is used frequently as a key factor in packaging materials, reached the lowest value for the LDPE/MgO-L (1:5 wt/wt) composite. The vapor transmission evaluated for that material is almost twofold lower than for neat LDPE. It should be underlined that the oxygen permeability of composite films was significantly affected by the microstructure in our composites, as LDPE/MgO-L (1:5 wt/wt) was characterized by the most uniform particle distribution and a lack of aggregate formation. The UV-barrier property of the composite films can be used as UV-screening films for food packaging application, especially for the best material, which again was the LDPE/MgO-L (1:5 wt/wt) composite. Therefore, we summarize that combining oxide and lignin via mechanical synthesis is a promising and practical direction to prepare novel compounds, which include bio-resource components. Based on our previous reports as well as the present study, after extensive studies of triple material composites LDPE/MgO-lignin, we confirm that the stoichiometric composition of hybrids and their excellent spread in polymer matrix are the key factors that influence the most important properties. Moreover, in our earlier studies, we confirmed that the mechanical as well as technological (weldability, thermoforming ability) properties are better than those of LDPE, which is the most popular food packaging material. Additionally, the optical properties changed notably for the hybrid films, and the anti-UV properties of the hybrid films are significantly improved by UV absorbance.

## Figures and Tables

**Table 1:** Percentage contributions of MgO-lignin (MgO-L) bio-filler into LDPE matrix in films extruded with a chill roll speed of 2 m/min.

Film Composition	Composition		
	Polymer Content (%) of Weight	Filler Content (%) of Weight	PE-g-MAH (%) of Weight
LDPE	100.0	-	-
LDPE/MgO LDPE/MgO-L (5:1 wt/wt) LDPE/MgO-L (1:1 wt/wt) LDPE/MgO-L (1:5 wt/wt) LDPE/Lignin	93.0	5.0	2.0

**Table 2:** Calorimetric parameters from first heating, cooling, and second heating scans for all materials.

Sample	T_m1_ (°C)	ΔH_m1_ (J/g)	X_c1_ (%)	T_c_ (°C)	T_m2_ (°C)	X_c2_ (%)
LDPE	114.5	110.7	37.78	95.8	114.5	40.36
LDPE/MgO LDPE/MgO-L (5:1 wt/wt) LDPE/MgO-L (1:1 wt/wt) LDPE/MgO-L (1:5 wt/wt) LDPE/Lignin	114.2	108.8	37.05	96.7	113.3	39.96
	115.8	104.9	35.79	95.9	114.3	39.33
	110.2; 115.8	104.2	35.55	96.0	113.6	40.22
	114.0; 116.4	114.4	39.04	96.6	114.0	37.57
	116.6; 114.3	118.7	40.50	96.9	113.0	41.36

Note: T_m1_, T_m2_–melting peak temperature at 1st and 2nd heating; ΔH_m1_–enthalpy of melting at 1st heating; X_c1_, X_c2_–crystallinity at 1st and 2nd heating; T_c_–crystallization peak temperature.

**Table 3:** Oxygen transmission rate (OTR) of neat LDPE film and composite films.

Sample	O_2_ Permeability	S.D.	Change
	(mL/m^2^/Day atm)		(%)
LDPE	779.0	5.1	-
LDPE/MgO LDPE/MgO-L (5:1 wt/wt) LDPE/MgO-L (1:1 wt/wt) LDPE/MgO-L (1:5 wt/wt) LDPE/Lignin	1292.6	118.2	rise 65
	920.3	54.8	rise 18
	1011.5	84.1	rise 29
	635.1	33.9	drop 18
	818.1	55.1	rise 5

**Table 4:** Water vapor transmission rate (WVTR) of neat LDPE film and composite films.

Sample	WVTR	S.D.	Change
	(g/m^2^/Day)		(%)
LDPE	8.16	0.05	-
LDPE/MgO LDPE/MgO-L (5:1 wt/wt) LDPE/MgO-L (1:1 wt/wt) LDPE/MgO-L (1:5 wt/wt) LDPE/Lignin	5.00	0.63	drop 38
	3.85	0.91	drop 52
	4.57	1.17	drop 43
	3.53	0.26	drop 56
	2.56	0.24	drop 68

## Data Availability

Data sharing is not applicable for this article.
